# Diagnostic Benefit of Simultaneous Capsule Endoscopy Using Two Different Systems

**DOI:** 10.1155/2018/9798546

**Published:** 2018-05-28

**Authors:** Seung Han Kim, Hyuk Soon Choi, Hoon Jai Chun, Eun Sun Kim, Bora Keum, Yeon Seok Seo, Yoon Tae Jeen, Hong Sik Lee, Soon Ho Um, Chang Duck Kim

**Affiliations:** Division of Gastroenterology and Hepatology, Department of Internal Medicine, Institute of Gastrointestinal Medical Instrument Research, Korea University College of Medicine, Seoul, Republic of Korea

## Abstract

**Background/Aims:**

Capsule endoscopy (CE) is a noninvasive test for diagnosing small bowel disorders. However, several studies reported that the CE-based visualization is suboptimal. This study, the first to use two CEs simultaneously, aimed at evaluating the diagnostic ability of dual CE.

**Methods:**

Dual CE procedures were prospectively conducted. All patients completed bowel cleansing 2 hours before examination. Subsequently, they simultaneously swallowed two capsules: MiroCam (IntroMedic, Seoul, Korea) and PillCam SB3 (Medtronic, Minneapolis, USA). We assessed the completeness and feasibility of small bowel examination and the detection rate of duodenal papilla and diagnostic yield.

**Results:**

Twenty consecutive patients who underwent complete small bowel examination with dual CE were enrolled in the study. The mean time of small bowel passage was 245 ± 99 min. Dual CE examination increased the duodenal papilla detection rate to up to 75% (versus PillCam SB3 alone (*P* = 0.031) and MiroCam alone (*P* = 0.063)) and overall diagnostic yield to up to 70% (*P* = 0.063) in comparison to single CE. Adverse events or electrical interference during data transmission between the two capsule endoscopes were not detected.

**Conclusions:**

In this study, we found that dual CE enhances diagnostic accuracy and could increase the diagnostic power of existing CE systems using simply applicable methods. This trial is registered with KCT0002541.

## 1. Introduction

Capsule endoscopy (CE) enables imaging of the gastrointestinal tract by transferring images wirelessly from a capsule endoscope to a data recorder through a sensing system. The introduction of CE provided a significant advancement in the diagnosis and management of small bowel diseases. The current diagnostic yields of CE in prior studies were 35% to 77% in patients with obscure gastrointestinal bleeding (OGIB) [1–8] and 55% to 83% in patients with Crohn's disease [9–12]. In spite of the revolutionized improvement in investigating the small bowel by CE, a considerable number of patients with small bowel diseases, such as OGIB, remain without a definitive diagnosis. Not only is the proper management of these patients not well known but it also is substantially challenging in clinical practice. Because CE is a noninvasive option and has been demonstrated to be more beneficial compared to other available approaches for the inspection of the small bowel, we speculated if different methods of CE could help these patients. To increase the examination's diagnostic yield, several methods have been proposed, with various results. A repeat CE has been indicated in patients with previously negative CEs to exclude missed findings. This repeat CE method has obtained additional positive findings in 49% of patients in a prospective study of 76 patients with continuous small bowel bleeding [13] and 35% of positive or suspected findings in a small prospective study [14]. Additionally, other studies whereby sequential application of capsule endoscopes was performed reported the detection of additional new lesions (8.3% to 19.6%) [15, 16] and presented no significant interference in electrical transmission between MiroCam (IntroMedic, Seoul, Korea) and PillCam SB CE (Given Imaging, Yokneam, Israel) [16]. However, these methods are time-consuming and require extensive radiological examination for additional capsule administration. In addition, the results from previous studies might not be fully concordant with real clinical outcomes.

CE can bypass about 30% of separate lesions, particularly in the proximal small bowel [17, 18]. This is due to gut motility and the fundamental technical limitations of CE. Although CE continues advancing in technical aspects and various types of devices are being developed, the efficacy of these devices is uncertain [17, 19].

Increasing the diagnostic yield of CE prior to specific interventions may have a far better impact on the treatment course [20]; this may also result in the overall reduction of medical expenditure.

This is the first clinical study to use two CEs simultaneously. In this pilot study, we performed simultaneous application of the two capsule endoscopes and evaluated the detection rate of duodenal papilla and diagnostic yield.

## 2. Materials and Methods

Patients indicated for CE, including those with OGIB, chronic diarrhea, and chronic abdominal pain, were included in the trial. All patients were between 20–80 years old. Patients suspected of having small bowel stenosis, swallowing difficulties, severe advanced respiratory disease, or cardiovascular or neuropsychiatric disease were excluded. Pregnant patients and those with implantable electronic devices were also excluded. All patients underwent esophagogastroduodenoscopy (EGD) and colonoscopy before the dual CE examination.

This study protocol adhered to the principles of the Declaration of Helsinki and was reviewed and approved ethically by the institutional review board of the Korea University Anam Hospital (Permit Number: ED16199). All patients provided a written informed consent at the time of enrollment.

The patients underwent fasting for 8 hours and completed bowel cleansing with 2 L of polyethylene glycol solution with ascorbic acid (Coolprep; TaeJoon Pharmaceuticals, Seoul, Korea) 2 hours prior to the examination [21, 22]. Subsequently, the patients swallowed two capsules, MiroCam (IntroMedic, Seoul, Korea) and PillCam SB3 (Medtronic, Minneapolis, USA), with a 5-minute interval. The swallowing order of the devices (MiroCam first versus PillCam SB3 first) was decided by randomization.

Examinations of the CE procedures were performed according to previously published guidelines [21, 22]. The findings of dual CE were assessed by a rate of 15 images per second. Two qualified and experienced CE reviewers interpreted the images taken by dual CE, with their findings being kept from each other. When the two reviewers have conflicting findings for the same patient, a third reviewer conducted clarification of the CE images for confirmation.

### 2.1. Statistical Analysis

Statistical analysis was performed using IBM SPSS Statistics for Windows, Version 20.0 (IBM Corp., Armonk, NY, USA). Completion rates and detection rates of the dual CE were analyzed using the McNemar test. Reading times of the two capsule endoscopes were compared using the Wilcoxon signed-rank test. The kappa value was used to evaluate the agreement between PillCam SB3 and MiroCam. In all tests, *P* values < 0.05 were considered statistically significant.

## 3. Results

### 3.1. Baseline Characteristics of Subjects

A total of 20 consecutive patients who underwent dual CE procedures were enrolled in the study. Among these patients, 12 (60%) were male and 8 (40%) were female; the mean age of the patients was 59.8 ± 14.4 (range 33–78) years. The indications for CE were as follows: OGIB in 15 patients (75%) and chronic abdominal pain or diarrhea in 5 patients (25%) (Table 1). All patients underwent preprocedure bowel preparation and swallowed the capsules without difficulty. Small bowel investigation was conducted on all dual CE procedures. The mean time of small bowel passage was 245 ± 99 min. The mean total operating times of PillCam SB3 and MiroCam were 14 h 1 min ± 125 min and 11 h 33 min ± 80 min (*P* < 0.05), respectively. Completion to the cecum of PillCam SB3 and MiroCam was 100% and 95%, respectively.

### 3.2. Detection Rate of CE

The duodenal papilla was identified in 9 (45%) patients with PillCam SB3 alone and 10 (50%) patients with MiroCam alone (Table 2). A comparative investigation of duodenal papilla detection rates in previous studies is presented in Table 3; the duodenal papilla detection rates ranged from 10.4% to 43.6%. The duodenal papilla detection rates of PillCam SB3 and MiroCam were 45% and 50% (*P* = 1), respectively. Dual CE examination increased the duodenal papilla detection rate up to 75% (versus PillCam SB3 alone (*P* = 0.031) and MiroCam alone (*P* = 0.063)). Significant findings were identified in 14 patients using dual CE procedures, leading to an overall diagnostic yield of 70% (55% for both PillCam SB3 and MiroCam (*P* = 0.063)). As previously presented, angiodysplasia of the small intestine is the most common positive finding, although mucosal ulcer, erosion, and polyps were also present. The agreement rate between PillCam SB3 and MiroCam was 70%, with a kappa value of 0.394 (*P* = 0.078) (Table 4). No significant adverse reaction in relation to dual CE examination was observed. In addition, electrical interference in data transmission between PillCam SB3 and MiroCam was not detected in any examination. However, short-term image disturbance disrupting the anterior visual field due to functional obstruction of the CE or illumination disturbance by the other device was observed.

**Figure 1:**
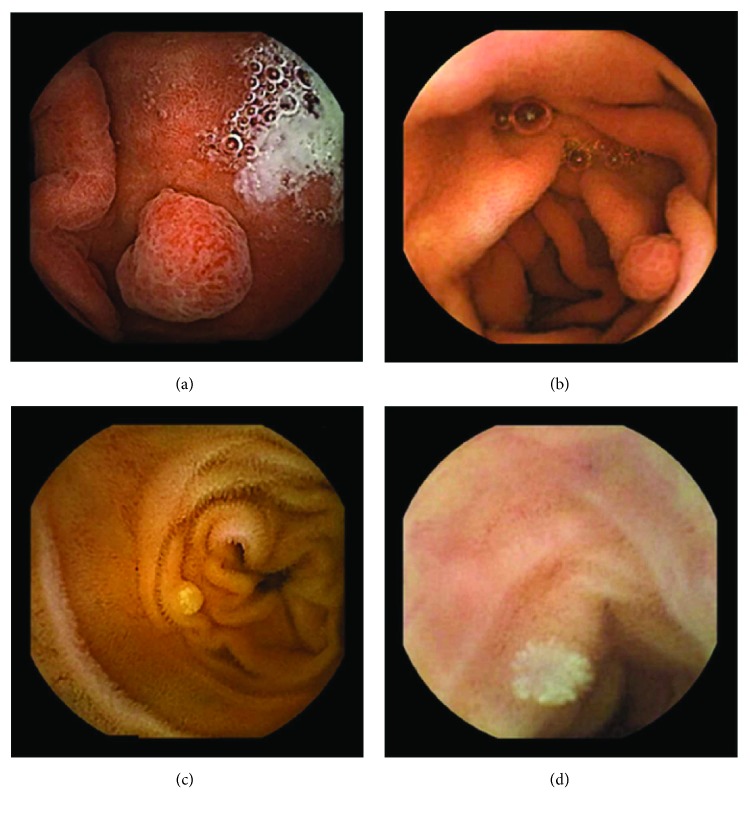
Examples of concordant cases. The MiroCam and PillCam SB3 revealed a polypoid mass in the proximal jejunum (a and b) of patient 2 and a polypoid mass in the distal ileum (c and d) of patient 7.

**Figure 2:**
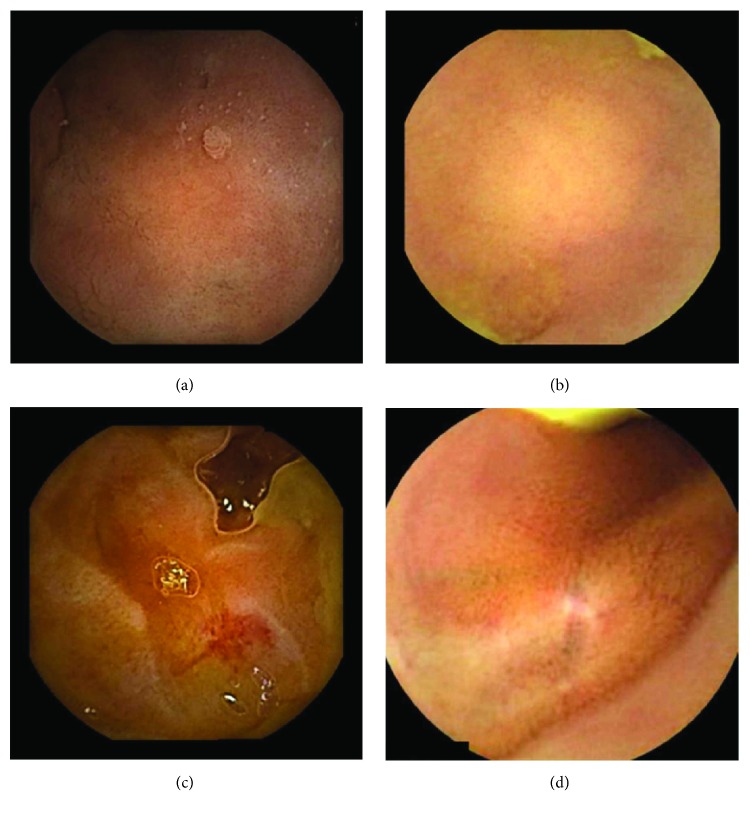
Examples of discordant cases. In patient 5, PillCam SB3 revealed a polypoid mass in the distal ileum (a), while MiroCam was not able to capture the lesion (b). In patient 6, the PillCam SB3 detected angiodysplasia in the jejunum (c), while the MiroCam could not (d).

In the examples of concordant cases, MiroCam and PillCam SB3 revealed polypoid masses in the distal ileum of the same patients (Figure 1). However, in the discordant cases, PillCam SB3 detected a polypoid mass in the ileum, while the MiroCam could not capture the lesion in the same patient (Figure 2). Additionally, PillCam SB3 was able to detect angiodysplasia in a patient, while the MiroCam could not.

**Figure 3:**
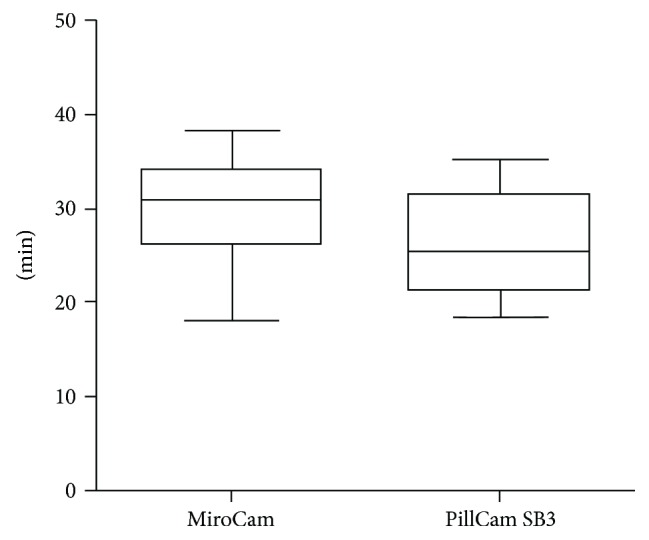
Comparison of reading times (MiroCam versus PillCam SB3 examination).

The mean reading time of the two devices were 26.3 ± 5.4 minutes for PillCam SB3 and 30.1 ± 5.5 minutes for the MiroCam (*P* < 0.05) (Figure 3).

## 4. Discussion

This prospective study demonstrated that dual CE is a safe and efficient tool for small bowel examination. To assess the ability of dual CE, we defined the duodenal papilla as the only landmark in the small bowel. As a result, the duodenal papilla was detected in more than 70% of the patients. To the best of our knowledge, this is the first clinical trial of simultaneous dual CE application. Previously, the concepts of repeat CE have been described. Although these methods offer additional findings compared to prior CE examination in patients with OGIB, they had disadvantages such as additional medical costs and being time-consuming [13, 16, 18]. In previous studies, the duodenal papilla detection rate ranged from 10.4% to 43.6%. In our study, a single CE examination demonstrated duodenal papilla detection rates of 50% and 45% for MiroCam and PillCam SB3, respectively. When dual CE was applied, the detection rate increased substantially, up to 75%, without significant adverse events. In addition, there was no significant interference of electrical transmission between the MiroCam and PillCam SB3 devices. MiroCam utilizes a new technology, the Human Body Communication, for data transmission; this is different from the 430 MHz radiofrequency telemetry system of PillCam SB3. In our study, we first tested the dual CE exam with two PillCam SB3 capsules, taken simultaneously. Interestingly, dual CE examination with two identical devices presented considerable visual interference throughout the procedure. In dual CE examination with two different devices (MiroCam and PillCam SB3), although temporary visual interference occurred due to physiological obstruction or illumination disturbance by the other device, it did not significantly decrease the visual field and diagnostic yield of dual CE.

We need to focus on the possible cost-effectiveness of dual CE exam in terms of total medical expenditure and patients' preferences. Although push enteroscopy and device-assisted enteroscopy (DAE), such as balloon-assisted enteroscopy, can directly evaluate the pathological lesions and perform procedures such as bleeding control or tissue sampling, their invasive nature hinders them from being conveniently performed as a first approach. Furthermore, current guidelines usually recommend DAE or push enteroscopy to be done as additional management for positive findings of CE [20, 23]. In that respect, dual CE exam could be a better alternative as a second-line diagnostic method for patients with small bowel diseases but without a definitive diagnosis, depending on the comorbidity, overall condition, or preference of the patients. Due to the high diagnostic accuracy and noninvasive nature of dual CE, it consequently has the potential for cost-effectiveness in terms of total medical expenditure and has a far better impact on patient management. In the future, the quality of CE exam might be improved to an extent such that the diagnostic yield could be increased dramatically [17]. However, in the present clinical practice, it might be sufficient to increase the diagnostic power of existing CE systems with applicable methods.

Although previous studies only compared the diagnostic yield of repeat CE, we also measured the detection rate of the duodenal papilla. There are no reliable criteria for CE examination; therefore, it is difficult to evaluate the actual diagnostic yield and sensitivity of CE. Additionally, the actual lesion visualized using CE does not necessarily coincide with the clinical outcome in many cases.

Dual CE exam did not increase unfavorable events, such as CE retention or small bowel obstruction. Prior to the application of dual CE, we need to exclude the potentially high-risk patients, such as patients with postoperative adhesions, with the use of abdominal CT scan or upper gastrointestinal series. Normally, dual CE could be performed safely in patients without complications or contraindications.

Our study has a few limitations. Because of its observational design, the number of enrolled patients was small for a pilot study, and furthermore no outcome data was presented, which might be altered after the dual CE procedure. However, a prospective study using both MiroCam and PillCam SB3 has not been previously conducted; therefore, we attempted to evaluate dual CE examination using these two capsules as they are readily available in Korea. Other capsule endoscopes such as the EndoCapsule (Olympus Medical Systems Corp., Tokyo, Japan) or OMOM capsule (Jinshan Science and Technology Co. Ltd., Chongqing, China) will require their own evaluation as part of a dual CE (e.g., MiroCam + EndoCapsule, MiroCam + OMOM capsule, or PillCam SB3 + OMOM capsule), given the variability of the availability of these devices in different countries.

This study demonstrated that dual CE is a safe and efficient tool for small bowel examination. Dual CE substantially increased the detection rate of duodenal papilla up to 75%, without significant adverse events. To clarify this result, further extensive research using dual CE is necessary. The occurrence of severe adverse events and significant interference of electrical transmission should be defined along with a large clinical trial. Additionally, alterations in treatment sequence or overall patient outcome after dual CE examinations should be investigated.

In conclusion, dual CE has a higher diagnostic accuracy than single CE. Dual CE could increase the diagnostic power of existing CE systems with the use of simply applicable methods.

## Figures and Tables

**Table 1:** Characteristics of patients and technical information on MiroCam and PillCam SB3 capsule endoscopes.

Number	Age	Sex	Indication	Total operating time of CE		Completion to the cecum		Significant findings or diagnosis on CE	
				MiroCam	PillCam SB3	MiroCam	PillCam SB3	MiroCam	PillCam SB3
1	59	M	Chr.Abd.pain	12:00:04	14:17:47	1	1	0	0
2	50	M	OGIB	07:30:42	14:50:20	1	1	Polyp	Polyp, erosion
3	66	F	OGIB	12:00:02	15:10:33	1	1	0	Ulcer, erosion
4	74	M	Chr.Abd.pain	11:57:22	14:43:57	1	1	0	0
5	67	F	OGIB	12:00:14	12:46:07	1	1	0	0
6	54	M	OGIB	12:00:26	15:34:43	1	1	Angiodysplasia	Angiodysplasia
7	63	M	OGIB	12:00:24	15:40:37	1	1	Angiodysplasia, polyp	Polyp
8	34	M	Chr.Abd.pain	12:00:14	15:12:39	1	1	0	0
9	61	F	OGIB	12:00:15	14:55:28	1	1	Angiodysplasia	0
10	64	M	OGIB	12:00:33	06:42:56	1	1	Angiodysplasia	0
11	50	F	OGIB	07:47:42	10:24:30	1	1	Polyp, erosion	Polyp, erosion
12	78	F	Chr.Abd.pain	12:00:03	15:07:23	1	1	0	0
13	56	M	Chr.Abd.pain	12:00:25	14:54:17	1	1	0	0
14	73	M	OGIB	12:00:22	12:11:14	1	1	Angiodysplasia	Angiodysplasia
15	73	M	OGIB	12:00:00	14:33:12	1	1	Angiodysplasia, ulcer	Ulcer, erosion
16	69	F	OGIB	12:00:00	13:26:19	1	1	Angiodysplasia, diverticulum	Erosion
17	71	F	OGIB	12:00:00	13:16:18	1	1	0	Angiodysplasia, erosion
18	33	M	OGIB	12:00:02	14:15:00	0	1	Angiodysplasia, erosion	0
19	65	F	OGIB	12:00:11	14:52:29	1	1	0	Ulcer scar, erosion
20	52	M	OGIB	12:00:05	14:53:12	1	1	Erosion	Angiodysplasia, erosion

Chr.Abd.pain: chronic abdominal pain; OGIB: obscure gastrointestinal bleeding.

**Table 2:** Duodenal papilla detection via the MiroCam and PillCam SB3 capsule endoscopes.

	MiroCam	PillCam SB3
Patient 1	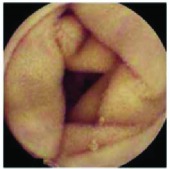	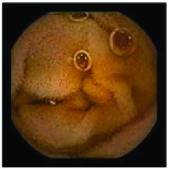
Patient 2	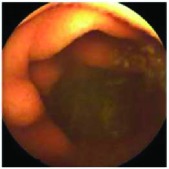	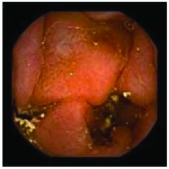
Patient 3	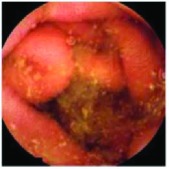	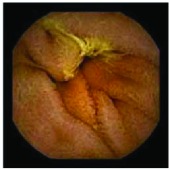
Patient 4	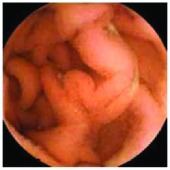	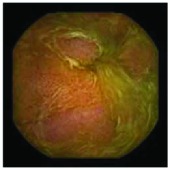
Patient 5	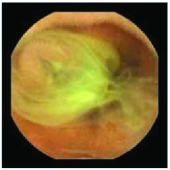	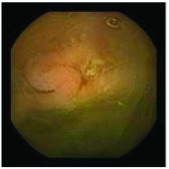
Patient 6	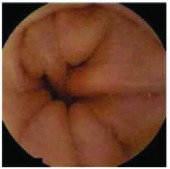	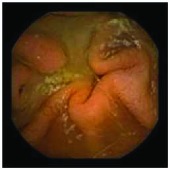
Patient 7	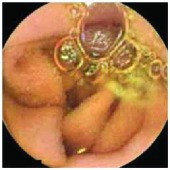	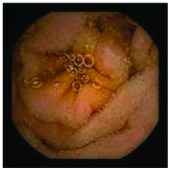
Patient 8	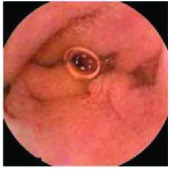	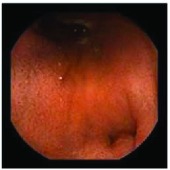
Patient 9	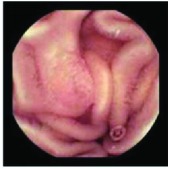	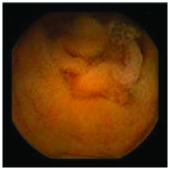
Patient 10	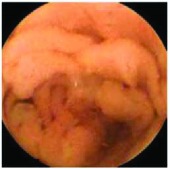	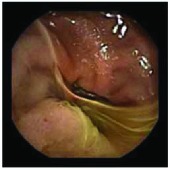
Patient 11	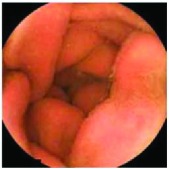	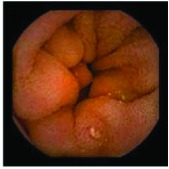
Patient 12	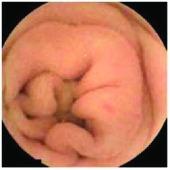	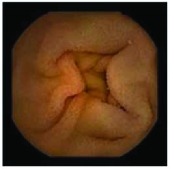
Patient 13	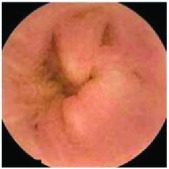	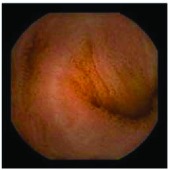
Patient 14	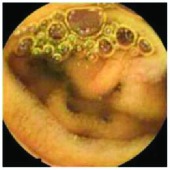	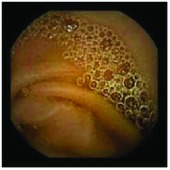
Patient 15	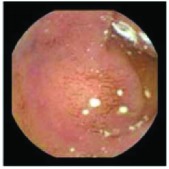	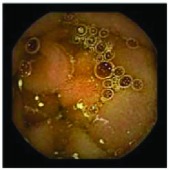
Patient 16	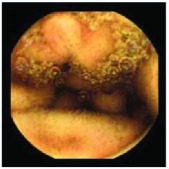	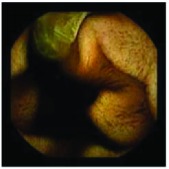
Patient 17	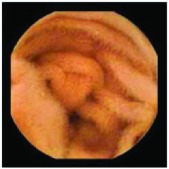	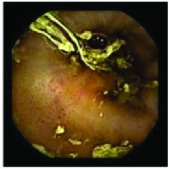
Patient 18	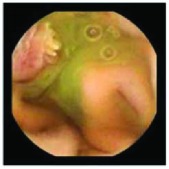	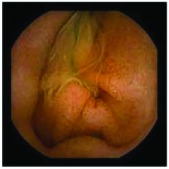
Patient 19	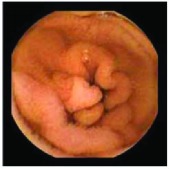	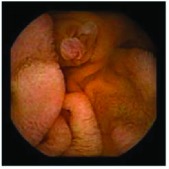
Patient 20	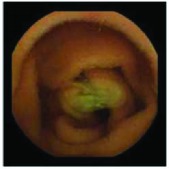	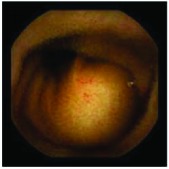

**Table 3:** Comparison of duodenal papilla detection rates.

Authors	Capsule	Study population (*n*)	Recording speed (frames/s)	Duodenal papilla detection (%)
Kong et al. [18]	M2A, Given Imaging	110	15	43.6
Clarke et al. [24]	SB1, Given Imaging	125	5	10.4
Nakamura et al. [25]	SB1, Given Imaging	96	10	18
Selby and Prakoso [26]	SB2, Given Imaging	50	n/s	18
Monteiro et al. [19]	SB3, Given Imaging	75	n/s	42.7

**Table 4:** Agreement between the MiroCam and PillCam SB3 results.

		PillCam SB		
		Negative	Significant abnormal	Total
MiroCam	Negative	6	3	9
	Significant abnormal	3	8	11
	Total	9	11	20

## References

[1] Lecleire S., Iwanicki-Caron I., Di-Fiore A. (2012). Yield and impact of emergency capsule enteroscopy in severe obscure-overt gastrointestinal bleeding. *Endoscopy*.

[2] Estévez E., González-Conde B., Vázquez-Iglesias J. L. (2006). Diagnostic yield and clinical outcomes after capsule endoscopy in 100 consecutive patients with obscure gastrointestinal bleeding. *European Journal of Gastroenterology & Hepatology*.

[3] Robinson C. A., Jackson C., Condon D., Gerson L. B. (2011). Impact of inpatient status and gender on small-bowel capsule endoscopy findings. *Gastrointestinal Endoscopy*.

[4] van Turenhout S. T., Jacobs M. A., van Weyenberg S. J. (2010). Diagnostic yield of capsule endoscopy in a tertiary hospital in patients with obscure gastrointestinal bleeding. *Journal of Gastrointestinal and Liver Diseases*.

[5] Yamada A., Watabe H., Kobayashi Y., Yamaji Y., Yoshida H., Koike K. (2012). Timing of capsule endoscopy influences the diagnosis and outcome in obscure-overt gastrointestinal bleeding. *Hepato-Gastroenterology*.

[6] Sidhu R., Sanders D. S., Kapur K., Leeds J. S., McAlindon M. E. (2009). Factors predicting the diagnostic yield and intervention in obscure gastrointestinal bleeding investigated using capsule endoscopy. *Journal of Gastrointestinal and Liver Diseases*.

[7] Albert J. G., Schülbe R., Hahn L. (2008). Impact of capsule endoscopy on outcome in mid-intestinal bleeding: a multicentre cohort study in 285 patients. *European Journal of Gastroenterology & Hepatology*.

[8] Min Y. W., Chang D. K. (2016). The role of capsule endoscopy in patients with obscure gastrointestinal bleeding. *Clinical Endoscopy*.

[9] Hara A. K., Leighton J. A., Sharma V. K., Heigh R. I., Fleischer D. E. (2005). Imaging of small bowel disease: comparison of capsule endoscopy, standard endoscopy, barium examination, and CT. *Radiographics*.

[10] Fireman Z., Mahajna E., Broide E. (2003). Diagnosing small bowel Crohn’s disease with wireless capsule endoscopy. *Gut*.

[11] Solem C. A., Loftus E. V., Fletcher J. G. (2008). Small-bowel imaging in Crohn’s disease: a prospective, blinded, 4-way comparison trial. *Gastrointestinal Endoscopy*.

[12] Eliakim R., Suissa A., Yassin K., Katz D., Fischer D. (2004). Wireless capsule video endoscopy compared to barium follow-through and computerised tomography in patients with suspected Crohn’s disease—final report. *Digestive and Liver Disease*.

[13] Viazis N., Papaxoinis K., Vlachogiannakos J., Efthymiou A., Theodoropoulos I., Karamanolis D. G. (2009). Is there a role for second-look capsule endoscopy in patients with obscure GI bleeding after a nondiagnostic first test?. *Gastrointestinal Endoscopy*.

[14] Bar-Meir S., Eliakim R., Nadler M. (2004). Second capsule endoscopy for patients with severe iron deficiency anemia. *Gastrointestinal Endoscopy*.

[15] Hartmann D., Eickhoff A., Damian U., Riemann J. F. (2007). Diagnosis of small-bowel pathology using paired capsule endoscopy with two different devices: a randomized study. *Endoscopy*.

[16] Kim H. M., Kim Y. J., Kim H. J. (2010). A pilot study of sequential capsule endoscopy using MiroCam and PillCam SB devices with different transmission technologies. *Gut and Liver*.

[17] Tontini G. E., Wiedbrauck F., Cavallaro F. (2017). Small-bowel capsule endoscopy with panoramic view: results of the first multicenter, observational study (with videos). *Gastrointestinal Endoscopy*.

[18] Kong H., Kim Y. S., Hyun J. J. (2006). Limited ability of capsule endoscopy to detect normally positioned duodenal papilla. *Gastrointestinal Endoscopy*.

[19] Monteiro S., de Castro F. D., Carvalho P. B., Moreira M. J., Rosa B., Cotter J. (2016). PillCam® SB3 capsule: does the increased frame rate eliminate the risk of missing lesions?. *World Journal of Gastroenterology*.

[20] ASGE Standards of Practice Committee, Gurudu S. R., Bruining D. H. (2017). The role of endoscopy in the management of suspected small-bowel bleeding. *Gastrointestinal Endoscopy*.

[21] Song H. J., Moon J. S., Do J. H. (2013). Guidelines for bowel preparation before video capsule endoscopy. *Clinical Endoscopy*.

[22] Ladas S. D., Triantafyllou K., Spada C. (2010). European Society of Gastrointestinal Endoscopy (ESGE): recommendations (2009) on clinical use of video capsule endoscopy to investigate small-bowel, esophageal and colonic diseases. *Endoscopy*.

[23] Enns R. A., Hookey L., Armstrong D. (2017). Clinical practice guidelines for the use of video capsule endoscopy. *Gastroenterology*.

[24] Clarke J. O., Giday S. A., Magno P. (2008). How good is capsule endoscopy for detection of periampullary lesions? Results of a tertiary-referral center. *Gastrointestinal Endoscopy*.

[25] Nakamura M., Ohmiya N., Shirai O. (2009). Advance of video capsule endoscopy and the detection of anatomic landmarks. *Hepato-Gastroenterology*.

[26] Selby W. S., Prakoso E. (2011). The inability to visualize the ampulla of Vater is an inherent limitation of capsule endoscopy. *European Journal of Gastroenterology & Hepatology*.

